# Supplementary figures and images for: Bulinus snails in the Lake Victoria Basin in Kenya: Systematics and their role as hosts for schistosomes
Source: PLoS Negl Trop Dis. 2023 Feb 10;17(2):e0010752. doi: 10.1371/journal.pntd.0010752 (PMC9949660; doi:10.1371/journal.pntd.0010752)

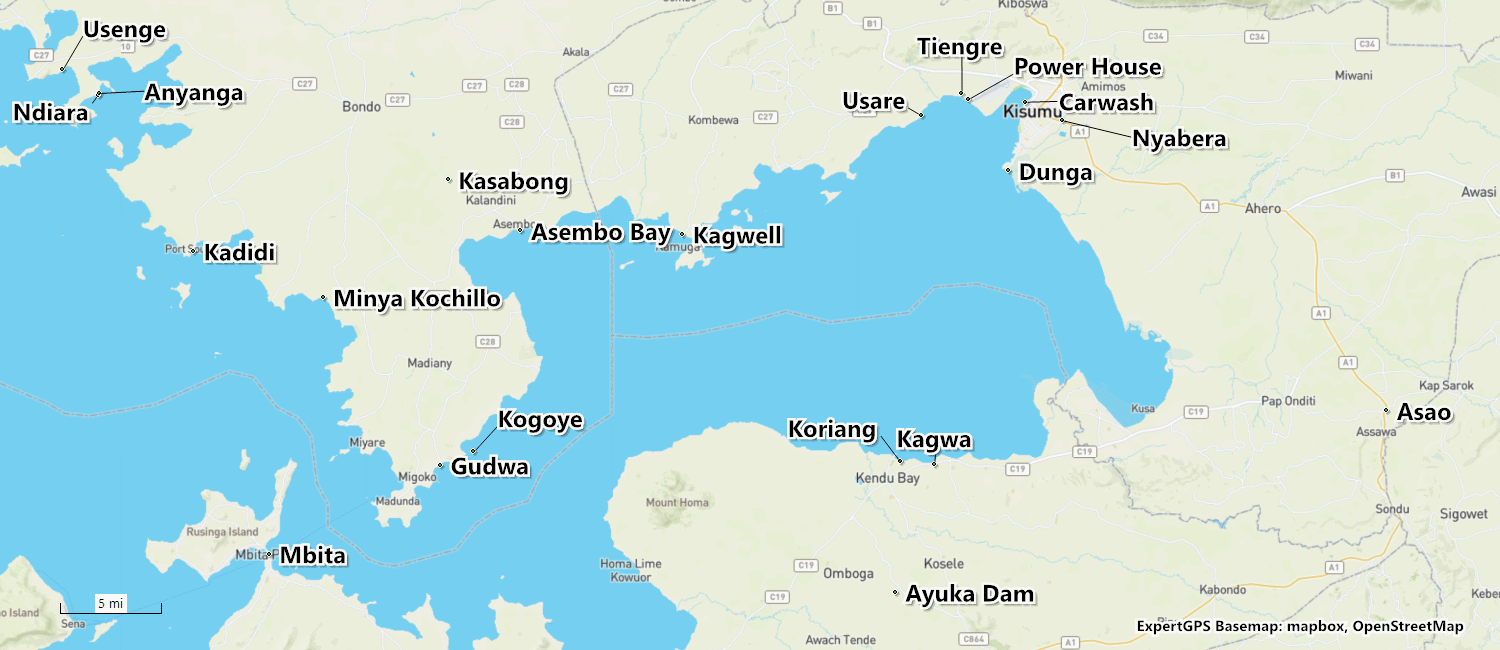

Supplement: S1 Fig — ExpertGPS Basemap of collection locations within the Lake Victoria Basin in Western Kenya for bulinid snails. Information regarding samples from these locations can be found in Tables 1 and S1. Base map and data from OpenStreetMap and OpenStreetMap Foundation. Base-layer retrieved from https://www.openstreetmap.org/relation/192798. (TIF) [file pntd.0010752.s001.tif]
