# Supplementary material for: Bulinus snails in the Lake Victoria Basin in Kenya: Systematics and their role as hosts for schistosomes
Source: PLoS Negl Trop Dis. 2023 Feb 10;17(2):e0010752. doi: 10.1371/journal.pntd.0010752 (PMC9949660; doi:10.1371/journal.pntd.0010752)
Supplement: Attachment — Submitted filename: Response to Reviewers signed.pdf [file pntd.0010752.s004.pdf]

CETI
Center for Evolutionary & Theoretical Immunology

December 11, 2022

Dear *PLOS Neglected Tropical Diseases* reviewer,

Please find enclosed our responses to reviewer comments. We found the reviewer comments to be very helpful in guiding us to create a more concise and impactful paper. For clarity, we highlighted reviewer comments and responded on each subsequent line in non-highlighted text. We also include a list of additional changes which were incorporated into the manuscript.

Final sentence of the abstract could be a little unclear for some readers. Define what pattern is referred to (species distribution? Schistosome compatibility? Lake vs non-lake?) and also what biological mechanisms are meant (i.e. are you referring to genes involved in resistance to schistosomes? Those that help with species ecology and for targeted snail control?). I fully appreciate that the authors want to be broad with this closing statement, but to me it does not provide enough information to make worthwhile including currently and I believe can be reworked to something much more impactful.

We reworked this sentence to specify that the pattern referred to is schistosome compatibility.

I really enjoyed reading both the introduction and the rich discussion, full of good insights and relating back to previous studies to summarize where we are at with the Bulinus genus in East Africa.

S1 Table and Table 1 – I find the way information is split across Table 1 and S1 Table a little bit frustrating. To make it easier for the reader, I believe an S1 table that has a summary of each site (i.e. rows), its coordinates, the waterbody type and number of Bulinus spp. (including break down of species) and number infected (and %) the number molecularly identified, summarized together would be more informative. Summarizing the data by site (i.e. rows), and breaking down the number of snails, and infected snails, collected within each species as columns would for me provide an easier reference than how laid out currently. Table 1 could then remain as is but habitat type, longitude and latitude could be removed as would be contained in S1 table, or another simplified summary table..

Altered the layout of table S1 as suggested to be arranged by location. Habitat and GPS were not removed from table 1 because there are sites (some from museum records) not included in table S1. Some collection location data is redundant between the two, but it is much clearer overall.

As an addition, it would also be good to have the temporal breakdown of when the snails were

collected, i.e. so that in future it can be used to identify snail abundance changes across seasons / years, as we know that this can be really important with the changing LVB. Although some of this temporal information is given in Table 1, it is not clear if this represents all the snails from collections from those sites or not. As all this information is there in the manuscript as is, this comment is more of a suggestion than a necessary change.

We agree that temporal data inclusion would be good, however, as this paper is more focused on defining bulinid species and the schistosomes they transmit, and we have plans to analyze this data with respect to rainfall and temperature data in the future, we would prefer not to lengthen this paper with inclusion of the temporal patterns.

A map Figure would be a wonderful addition to this manuscript to help represent where collections were made, the distance between sites, and where species were found to related to phylogenetic analysis. Would be great in the manuscript or even as a supplementary figure.

Agreed and added as a supplemental figure

Line by line comments

52 – Maybe a brief mention of S. mansoni group and Biomphalaria spp. presence?

Excellent suggestion. However, the author summary section is at its word limit and therefore this was not incorporated

59 – ‘DNA sequence based’

Changed as suggested

59 – ‘shed from infected snails’ - to clarify that looking at patent infections only.

Changed as suggested

97 – ‘includes 9 species: ..’

Changed as suggested

130 – change in reference style?

Reference style altered to match the rest of the manuscript

131 – think this paragraph should be attached to previous one to lead on from the end of the last paragraph ‘i.e. Previously… More recently..’ – since not starting a ‘new’ point.

Changed as suggested

131-137 – Not sure however if all this discussion necessary related to hybrids here. Suggest simplifying by removing last two sentences.

Removed the second to last sentence – kept the last sentence to keep the point that genetic compatibility may be partially dependent on schistosome genetic compatibility

139 – From reading rest of paper – doesn't seem that too much focus is given to 'other' trematodes outside schistosomes except for morphologically identifying to genus and brief part in results 353-358 – therefore I would reword this sentence to make clear this is really focusing on Bulinus and schistosomes, with some insight into trematodes too.

Sentence reworded to emphasize that the primary purpose of the paper is *Bulinus-schistosome* relations but includes some insight into non-schistosome trematode relations.

144 – for the readers ease, would be nice to have this list split up into the species groups they represent too?

Edited as suggested

165 – Sentence to use in reworking final abstract sentence?

Used in to rework final abstract sentence

171 – Gives the impression that localities are specifically defined in S1 Table, yet they are not really, just names This is provided in the my general comment above, but you could provide long / lat here in S1 table. But another suggestion might be just to include a summary table by site listening the name, coordinates, water body type and number of Bulinus spp. collected?

Table S1 GPS locations added

172 – Collections span from Jan 2014 to ?? Could specify here for this study at least, even if collections are continuing.

Changed as suggested

178 – Could it be more specifically stated how this combined 150m was achieved, or better, point to it in reference cited on line 173 i.e. Mutuku 2019 if it is contained in here

Removed description and instead referenced methods as described by Mutuku et al 2019

190 – Why the S. haematobium have collected from humans here is not clear. Can see from later in paper it is to compare with those shed from Bulinus in phylogenetic analysis. Worth mentioning that here in my opinion to be clear, as my thoughts were that experimental exposures may be taking place. Would be great to do challenges of the Lake Bulinus with schistosomes in the future.. Also could add, how many miracidia collected from x number of individuals?

Added rationale for *S. haematobium* collection from humans. We agree that experimental infections should be done and we have set up lab-reared colonies of the relevant *Bulinus* species. We are currently working to optimize our culture and exposure conditions, including to overcome the effects of chaetogaster infestations that develop on lab-reared bulinids and influence miracidium success. Total numbers of miracidia collected in the pooled samples were not determined.

213 – Could it be specified, maybe in table 1 – when alternative COR722b primers were used for amplification? Was this due to sequence diversity in particular species? Could be useful for reference in future studies.

In some cases, for reasons inapparent to us, and as is a common experience for others, some samples simply did not permit amplification with our original primers, so we used COR722b as an alternative, or this primer was used to enable us to fill in incomplete sequences. As there was no obvious pattern to why certain reactions did not work with the original primers, we think it best to simply mention in the materials and methods that this was an approach used rather than indicate which particular specimens for which it was used.

223 – were individual cercariae therefore removed from pooled ethanol preserved specimens?

Yes, edited for clarification

248 – references to associated studies could be included for the genbank accessioned used.

References added

261 – provide accession numbers for sequences in current study here too?

Referenced the tables in which the accession numbers can be found in

270 – Can you list the number of specimens identified to each species / species group in the main text. Also not clear to all readers in table S1 which parts represent species groups and which species – I presume B. forskalii listed in S1 Table is species group and not representing species alone, as must include the B. scalaris identified as noted in table 1 and later in manuscript? Denote that B. truncatus / tropicus group and forskalii group are therefore identified to species group level in text and in S1 table (unless I am misinterpreting?).

Number of specimens identified to species or species group has been added to text. Table S1 has been edited to convey that *B. forskalii* and *B. trunc/trop* group members were identified to the species group level and not species level in that table. Representative specimens were of course identified to species as indicated later in the paper.

272 – Highest / lowest S. haematobium prevalence observed from where? Of interest and could be mentioned in main text here briefly?

Added as suggested

289 – Denote in table and legend which samples from archived specimens? '*' i.e. B. nasutus?

Denoted as suggested

320 – As for Table 2 – could be good practice to include references to the reference sequences used in the phylogenetic tree? See earlier point in methods too.

References for reference sequences were added to the methods section after accession numbers are listed.

338 – Reference for Indoplanorbis sequences in Figure?

Accession numbers and references to associated studies can be found in the methods section

348 – Still would be interested to see how schistosome infections vary over time or specific sites mentioned in text. Would help highlight details in Table S1 regarding sites with lots of infected snails. No temporal detail for snail collections of infections included currently, but I believe could easily be added.

As noted above, we are currently compiling temporal data for a larger study including additional species, temperatures, rainfall, etc. across a number of habitats.

433 – Last sentence here seems a bit of a stub – understand where going with this paragraph in saying one might consider these a very wide complex of species, but could this paragraph be reworked to make more clear?

Moved to end of previous paragraph – It seems to read more smoothly now.

454 – Bulinus in full at sentence start.

Changed as suggested

545 – this attribute – can it be expanded on, hypothesized just in a few words? i.e. genetic resistance in snails or something else? Could this also be related to what is lead into the discussion in line 556 onwards?

Specified that the attribute is natural resistance to Sh infection
------------------

Additional changes:

25: removed "infecting animals" to keep abstract at 200 words.
Author summary: altered for clarity.
164-167: Removed "Ex" from before species names for clarity.

Center for Evolutionary & Theoretical Immunology

176: added "of compatibility" to clarify which pattern is being referred to.

209-210: clarified the source of miracidia and added "and used for phylogenetic analyses and comparisons with schistosomes shed from infected snails" to clarify the purpose of miracidia collection.

259: Added "1%" to specify what percent agarose was used.

275-276: added "GenBank accession numbers for sequences provided in this study can be found in Tables 1 and 3." to clarify where accession numbers provided by this study can be found.

291: added reference to S1 Fig

292: Added the word "initially" for clarification

298-299: added sentence "Further sequence-based specifications of species identities for both bulinids and schistosomes are found below."

Table 1: Removed redundant "LV" designations. Added P= pond to legend for consistency. Made dates consistent with YYYY-MM-DD style.

Fig 1 and Fig 2: altered to fix an identified mistake.

All figures removed for final submission

Table 2: Caption edited

375: Section title altered to be consistent with other titles

393: Changed font to appropriate heading size.

Table 3: Removed redundant "Kenya" designations. Added missing GPS coordinates.

445-446: added "with multiple lineages represented in Kenya alone" and an additional citation to reflect recent work related to this manuscript.

460: Removed word "hosts" because it was redundant

470-471: Added sentence "More recently Zhang *et al.* [46] assembled the mitogenome of a *B. ugandae* sample from Lake Victoria." with associated citation to reflect recent work on *B. ugandae* in this study area.

533: Altered phrasing for clarity.

540: Altered phrasing for clarity.

References: Were edited to match journal style (scientific names italicized etc.)

Many thanks for your careful consideration and evaluation of our work.

Caitlin Babbitt

Digitally signed by Caitlin Babbitt
DN: cn=Caitlin Babbitt, o, ou, email=cbabbitt@unm.edu, c=US
Date: 2022.12.16 09:30:04 -07'00'

Caitlin R. Babbitt
1 University of New Mexico
MSC03 2020
Department of Biology
University of New Mexico
Albuquerque, New Mexico 87131-0001
cbabbitt@unm.edu
